# Case report: Anomalous systemic drainage of ileocolic veins (veins of Retzius) causing pelviureteric junction obstruction

**DOI:** 10.4103/0971-3026.41838

**Published:** 2008-08

**Authors:** Praveen R Maheshwari, Omran Shammam

**Affiliations:** Department of Radiology, Tripoli Medical Center (TMC), Tripoli, Libya

**Keywords:** Ileocolic vein, pelviureteric junction obstruction, veins of Retzius

## Introduction

Pelviureteric junction (PUJ) obstruction can be due to an intrinsic neuromuscular abnormality or due to extrinsic compression. The most common extrinsic cause of PUJ obstruction is an aberrant lower pole renal artery.[1] PUJ obstruction due to lienorenal collaterals has also been reported.[2] However, to the best of our knowledge, PUJ obstruction due to the veins of Retzius has not been reported in the literature. The veins of Retzius are important as they can be injured during right hemicolectomy and because they may provide a pathway for the hematogenous spread of colon cancer. Based on our case report, we would like to stress the importance of recognizing the veins of Retzius as a potential cause of PUJ obstruction which, if unrecognized, could lead to significant vascular complications, including hemorrhage and compromise of the venous return of the colon.

## Case History

**Figure 1:**
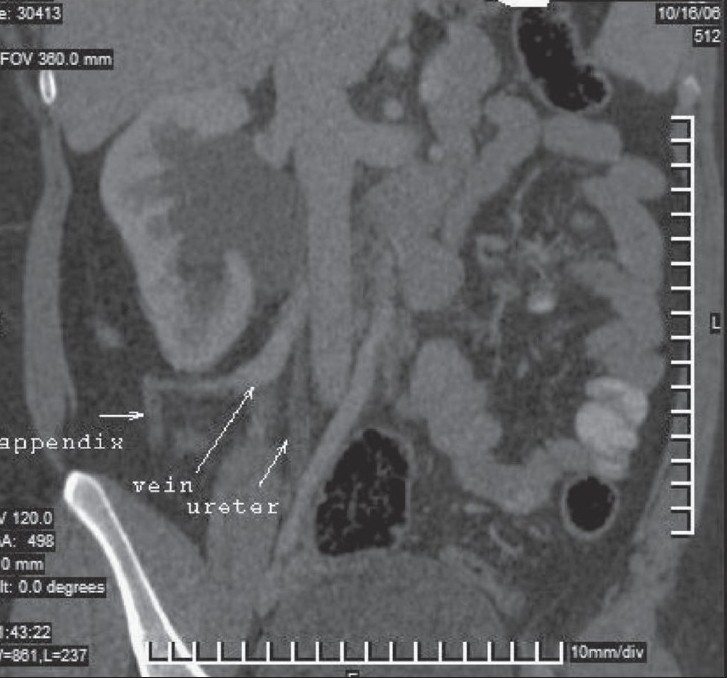
Oblique coronal multiplanar (MPR) image shows the ileocolic vein (vein of Retzius) draining into the IVC and causing PUJ obstruction

**Figure 2:**
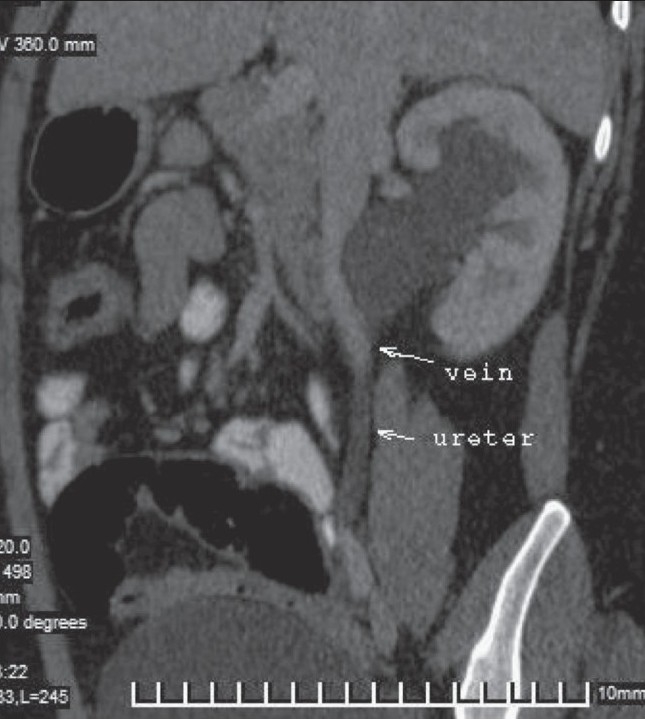
Oblique sagittal MPR image shows the relationship of the ileocolic vein to the PUJ

**Figure 3:**
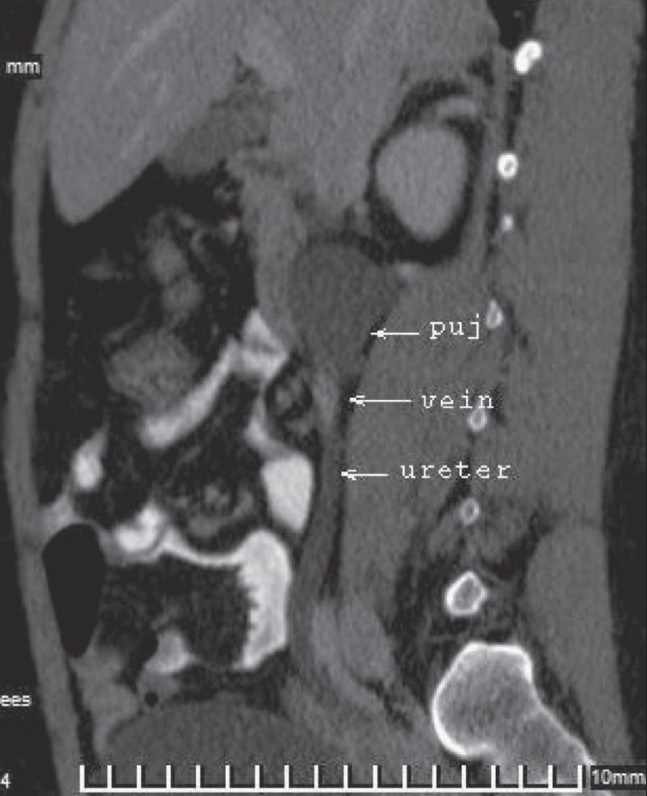
Sagittal MPR image shows the ileocolic vein coursing along the anterior aspect of the PUJ

**Figure 4:**
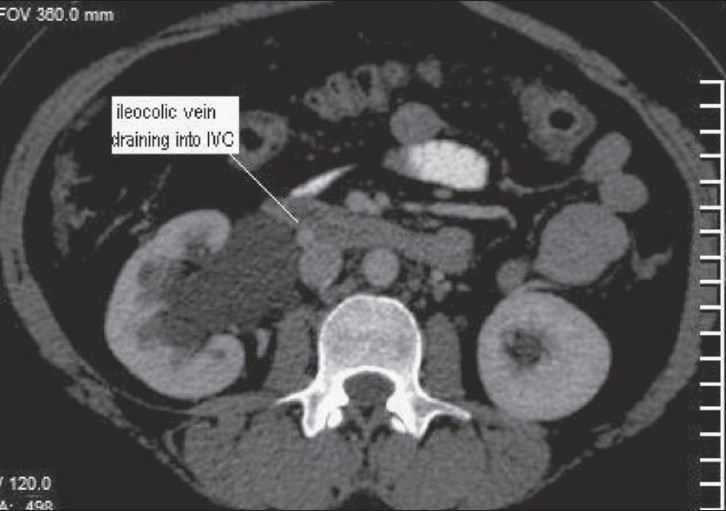
Axial image shows the ileocolic vein draining into the anterior aspect of the IVC

A 26-year-old woman presented with complaints of intermittent pain in the right flank for one year. USG revealed right hydronephrosis without hydroureter. There was no evidence of a calculus. The left kidney was normal. CT scan revealed the right hydronephrosis ending abruptly at the pelviureteric junction; there was a normal caliber ureter [Figure 1]. No intraluminal cause of obstruction could be seen on the CT scan. The ileocolic vein was seen coursing along the anterior aspect of the PUJ [Figures 2 and 3] and draining directly into the IVC [Figure 4]. No other vascular abnormality could be identified on the CT scan. There were no signs of liver cirrhosis. In view of these findings, an open surgical pyeloplasty was performed. The patient was symptom free after surgery.

## Discussion

PUJ obstruction can be due to intrinsic or extrinsic causes. Congenital PUJ obstruction is mostly due to an intrinsic neuromuscular abnormality.[1] Crossing renal vessels are the most common extrinsic cause of PUJ obstruction. A vein of Retzius causing PUJ obstruction has not been reported earlier.

The exact role of crossing vessels in the etiology of PUJ obstruction is a matter of debate; many believe that they are only incidental findings.[3] This has been disputed in some of the recent literature, which has shown that in some patients, the underlying ureter is entirely normal and surgical relocation of the vessel alone has been therapeutic.[3] Whether there is a cause and effect relationship or not, there is no controversy regarding the importance of the identification of these vessels before deciding on the choice of therapy (open pyeloplasty *vs* endopyelotomy). Endoscopic treatment has a 90% success rate, which drops to 40% in the presence of crossing vessels.[4] Unrecognized crossing vessels can also result in significant hemorrhage during endopyelotomy. Therefore, accurate preoperative imaging is crucial for appropriate treatment planning.[4] CT scan is an accurate and noninvasive method of demonstrating crossing vessels in PUJ obstruction.[2] 

The anastomoses between the branches of the superior or the inferior mesenteric veins and the IVC are called veins of Retzius. Anastomoses between the portal venous system and the systemic venous circulation can exist even under normal conditions. Ibukuro *et al.* identified veins of Retzius at CT arterioportography in approximately an equal percentage of patients with and without liver cirrhosis.[5] In contrast to other portosystemic shunts, even in patients with portal hypertension, the veins of Retzius are often not dilated and hence not well recognized. An ileocolic vein draining into the IVC or the right renal vein through the right gonadal vein is the most frequently demonstrated pathway of the veins of Retzius.[5] 

In our case, an ileocolic vein was found draining directly into the IVC, which is the least common pathway of the veins of Retzius, being seen only in a very small percentage of patients. In the cases of Ibukuro *et al.*[5] the ileocolic vein was shown entering the right side of the IVC, whereas in our case the ileocolic vein was found draining into the anterior aspect of the IVC, coursing along the ventral aspect of the PUJ and causing PUJ obstruction.

In conclusion, we believe that all cases of PUJ obstruction must be carefully evaluated for any crossing vessel.
